# Designing n‑Type Thermogalvanic TEMPO-Substituted Polyacrylamide via Conformational Entropic Modulation

**DOI:** 10.1021/acsmacrolett.5c00762

**Published:** 2026-01-22

**Authors:** Ching-Chieh Hsu, Kohei Ishigami, Ryo Shirakawa, Hiroyuki Nishide, Kenichi Oyaizu, Cheng-Liang Liu

**Affiliations:** † Department of Materials Science and Engineering, National Taiwan University, Taipei 10617, Taiwan; ‡ Department of Applied Chemistry, 33561 Waseda University, 3-4-1 Okubo, Shinjuku, Tokyo 169-8555, Japan; § Research Institute for Science and Engineering, 13148 Waseda University, 3-4-1 Okubo, Shinjuku, Tokyo 169-8555, Japan; ∥ Institute of Polymer Science and Engineering, National Taiwan University, Taipei 10617, Taiwan; ⊥ Advanced Research Center for Green Materials Science and Technology, National Taiwan University, Taipei 10617, Taiwan

## Abstract

This work pioneers the use of TEMPO-substituted polyacrylamide (PTAm) for n-type thermogalvanic (TG) systems, uniquely harnessing redox-induced conformational entropy changes to enhance the thermoelectric performance. Through the electrochemical oxidation of low-molecular-weight PTAm, which is initially water-insoluble, a water-soluble polyelectrolyte (ox-PTAm) is formed, containing both TEMPO and oxoammonium species, as indicated by cyclic voltammetry. The redox transitions induce conformational entropy changes, which are corroborated by electrochemical and thermoelectric measurements, leading to an observed n-type thermopower (α) of −0.76 mV K^–1^. A maximum power output of 1.18 mW m^–2^ K^–2^ is achieved under a thermal gradient of 3.8 K. This work highlights the potential of entropy modulation in redox-active polymers as a strategy for advancing organic thermoelectric materials targeting low-grade waste heat recovery.

## Graphical abstract

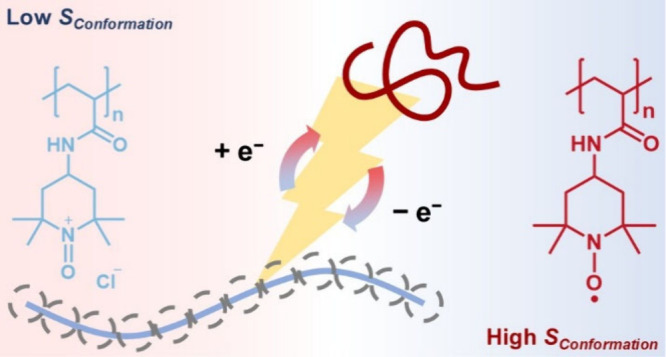

The global energy crisis^1^ remains a pressing challenge, driving demand for sustainable energy solutions. Among various renewable energy sources, low-grade waste heat (<100 °C)^2–5^ has attracted increasing attention due to its widespread availability and underutilization. Conventional thermoelectric generators, such as those based on the organic Rankine cycle,^6–9^ offer a route to recover waste heat. However, their dependence on bulky architectures and moving components limits their applicability in compact or wearable devices.^10–12^ Thus, the development of stationary thermoelectric materials^13–17^ is essential for efficient low-grade heat harvesting. Thermogalvanic (TG) materials^18–27^ represent a promising alternative for converting low-grade thermal energy into electricity. These systems typically exhibit high thermopower (|α| ∼ 1–10 mV K^–1^), significantly outperforming conventional electronic conducting and semiconducting thermoelectric materials (|α| ∼ 1–100 μV K^–1^).^28–31^ TG cells operate by exploiting the temperature dependence of Faradaic redox potentials: when a temperature gradient is applied across the electrolyte, the redox potential difference between the hot and the cold electrodes generates a voltage across the cell. The thermopower (α) of a TG cell can be derived by following equations:

1 $$\Delta G_{\mathrm{Hot}}=-nFE_{\mathrm{Hot}}=\Delta H-T_{\mathrm{Hot}}\Delta S_{\mathrm{red}}$$

2 $$\Delta G_{\mathrm{Cold}}=-nFE_{\mathrm{Cold}}=\Delta H-T_{\mathrm{Cold}}\Delta S_{\mathrm{red}}$$

It may be noted that the reaction entropy and enthalpy at the hot and cold ends should be approximately the same, as the temperature gradient is not large enough to cause a significant deviation. Subtracting eq 2 from eq 1 yields the α expression:

3 $$\alpha =-\frac{\Delta E}{\Delta T}=-\frac{E_{\mathrm{Hot}}-E_{\mathrm{Cold}}}{T_{\mathrm{Hot}}-T_{\mathrm{Cold}}}=-\frac{\Delta S_{\mathrm{red}}}{nF}$$

where *E*_Hot_ and *E*_Cold_ represent the redox potential at hot side and cold side, respectively; *T*_hot_ and *T*_cold_ for the corresponding temperatures; Δ*S*_red_ for the reduction reaction entropy; *n* for number of electrons transferred; *F* for Faradaic constant. Notably, eq 3 mirrors the temperature coefficient commonly used in electrochemistry with an added negative sign to align with the Seebeck coefficient convention in electronic thermoelectrics.

From eq 3, enhancing Δ*S*_red_ is a fundamental strategy for improving the thermopower in TG systems. Several approaches, such as solvation shell engineering, have been proposed to increase Δ*S*_red_. Zhou et al. demonstrated that a viologen-substituted copolymer,^32^ poly­(NIPAM-*co*-*N*-(2-acrylamide ethyl)-*N*′-*n*-propylviologen) (PNV), exhibited a significant structural entropy change upon redox switching within a specific temperature range. This behavior originated from the redox-induced modulation of the polymer’s lower critical solution temperature (LCST), leading to distinct conformational transitions that greatly enhanced Δ*S*_red_. Despite this promising approach, the main challenge of this strategy is the limited operating-temperature window. The enhanced Δ*S*_red_ can be observed only between the LCST of oxidized/reduced polymer. Moreover, the role conformational entropy without phase transitions, which is irrelevant with the operating temperature, in TG polymer remains underexplored. In this work, we present a (2,2,6,6-tetramethylpiperidin-1-yl)­oxyl (TEMPO)-substituted polyacrylamide (PTAm) as a redox-active polymer for TG applications to eliminate the temperature window constraints allowing for greater flexibility. The fast redox kinetics of the TEMPO radical enables efficient charge transfer ability toward electrode, resulting in a high output current. Additionally, the conformational entropy change of polymer also enables n-type TG behavior. This study demonstrates a structure-induced entropy enhancement strategy for TG aqueous cells, achieving a thermopower of α = −0.76 mV K^–1^ and a maximum power output normalized by the temperature square (*P*_max_/Δ*T* ^2^) of 1.18 mW m^–2^ K^–2^, thereby advancing the prospects of low-grade heat harvesting.

The synthesis of TEMPO-substituted polyacrylamide (PTAm) is described in detail in the Supporting Information. Briefly, the *N*-(2,2,6,6-tetramethylpiperidin-4-yl) acrylamide (TAm) monomer was synthesized^33^ by the reaction between acryloyl chloride (2.4 mL, 296 mmol) and 4-amino-2,2,6,6-tetramethylpiperidine (13 mL, 7.48 mmol). Subsequently, the TAm monomer (1.05 g, 5 mmol) was polymerized using 2,2′-azobis­(2-methylpropionitrile) (AIBN, 16.5 mg, 0.1 mmol) as the radical initiator. The polymerization product was then subjected to chemical oxidation with an excess of *m*-chloroperoxybenzoic acid (mCPBA), converting the pendant piperidine groups into TEMPO radicals, as illustrated in Scheme 1 (radical concentration of 83%, as described in Supplementary Note 1, and the ^1^H NMR spectrum of PTAm precursor was shown in Figure S2 in the Supporting Information). Gel permeation chromatography (GPC) revealed that the synthesized PTAm had a number-average molecular weight (*M*_n_) of 3.8 × 10^4^ with a polydispersity index (PDI) of 1.9 (Figure S3).

**Scheme 1:**
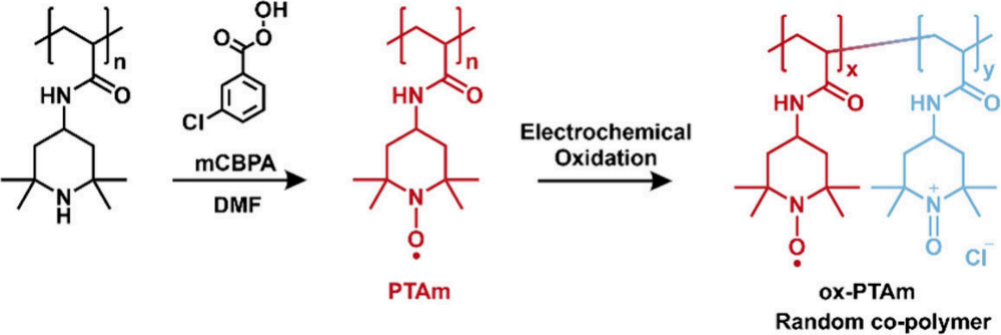
Schematic Representation of the Oxidation of 2,2,6,6-Tetramethylpiperidine-Substituted Polymer to PTAm^a^

a The oxidized PTAm (ox-PTAm) was obtained via electrochemical oxidation of PTAm.

As previously reported in the literature, PTAm synthesized in this work remains water-insoluble even at elevated temperatures. To probe its electrochemical behavior, PTAm coated on carbon cloth was evaluated by cyclic voltammetry (CV) in a U-type cell (PTAm|3 M KCl_(aq)_||3 M KCl_(aq)_|Pt), as shown in Figure S4. Over successive cycles, the both anodic and cathodic current gradually decreased, and the anolyte turned yellow, indicating the dissolution of oxidized PTAm (ox-PTAm) into the aqueous phase, which differs from previous reports on high molecular weight PTAm (*M*_w_ ∼ 10^6^, measured from GPC).^34,35^ This solubilization is attributed to the combined effects of the relatively low molecular weight and the ionic nature of the ox-PTAm. To verify the presence of the TEMPO moiety in solution, CV measurements were performed at varying scan rates (200–400 mV s^–1^), as shown in Figure 1a. At 200 mV s^–1^, sharp redox peak were observed at an anodic peak (*E*_pa_) = 0.706 V and a cathodic peak (*E*_pc_) = 0.646 V, yielding a formal potential (*E*_formal_) = 0.676 V. The peak-to-peak separation (Δ*E*_p_) increases only slightly from 60 mV at 200 mV s^–1^ to 68 mV at 400 mV s^–1^, indicating rapid and reversible electron transfer between the TEMPO moieties and the Pt electrode. Therefore, insoluble PTAm can thus be converted electrochemically to water-soluble ox-PTAm, enabling redox activity in aqueous environments. Moreover, the linear behavior observed in Figure 1b suggests the diffusion-controlled electrochemical behavior of ox-PTAm. Electrochemical impedance spectroscopy (EIS, Figure S5) further revealed typical electrochemical behavior with a high-frequency semicircle corresponding to charge transfer resistance (*R*_ct_) and a low-frequency linear tail associated with Warburg diffusion. From the Nyquist plot, the *R*_ct_ = 47.0 Ω were extracted using a Randles circuit model fitting. The heterogeneous electron transfer rate constant (*k* ^0^) was estimated using the following equation:^36^

4 $$k^{0}=\frac{RT}{n^{2}F^{2}ACR_{\mathrm{ct}}}$$

where *R* is the gas constant, *A* is the electrode area, and *C* is the redox species concentration. Although the concentrations of the oxidized and reduced species were not determined, assuming equal oxidized and reduced species concentrations (*C*_ox_ = *C*_red_), the *k* ^0^ of water-soluble ox-PTAm was estimated to be approximately 7.1 × 10^–3^ cm s^–1^, indicating the fast reaction kinetics between TEMPO/oxoammonium redox couple and Pt electrode, which is favorable for TG applications. Although the estimation relies on assumptions regarding redox concentrations, the small Δ*E*_p_ in CV measurements supports the conclusion of excellent reaction kinetics.

**Figure 1:**
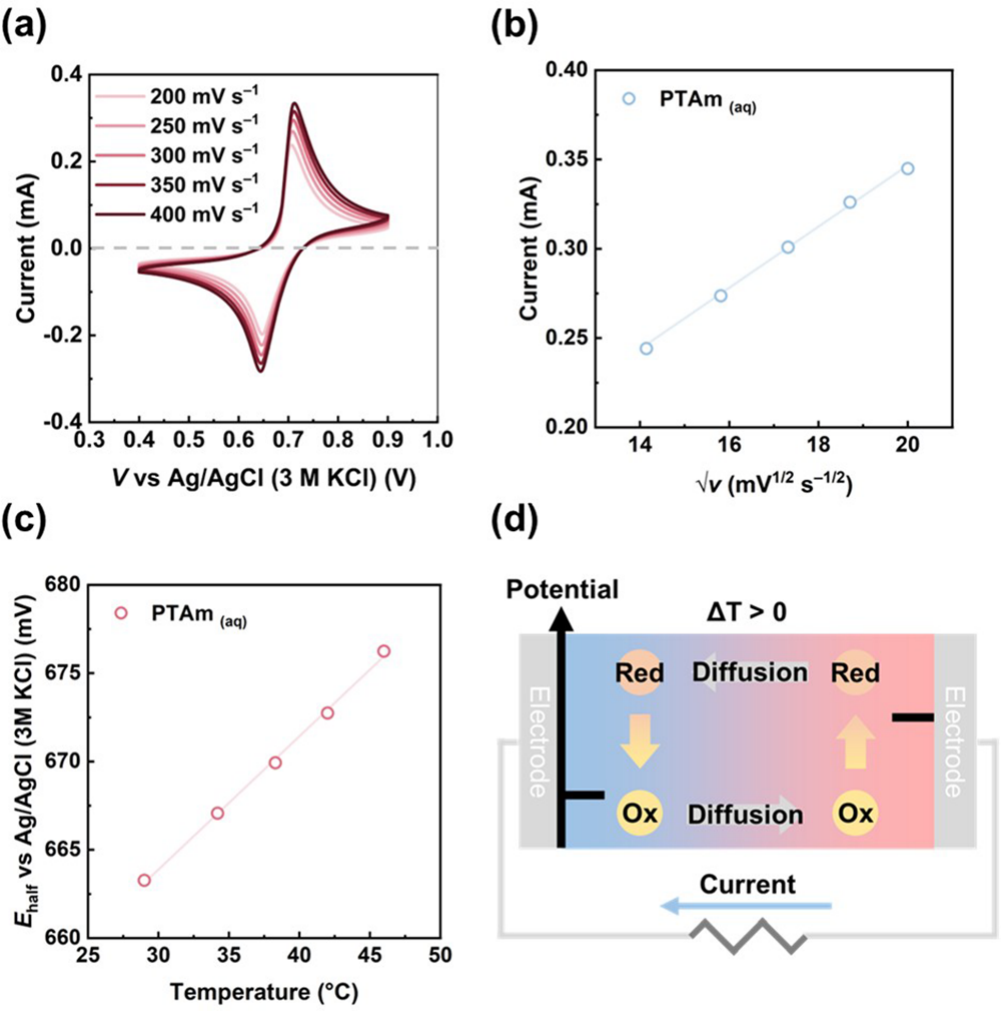
(a) CV curves of water-soluble ox-PTAm at scan rates ranging from 200 to 400 mV s^–1^. (b) Peak current in CV as a function of root of scan rate. (c) Half-cell potential (*E*_half_) as a function of temperature. (d) Schematic illustration of the n-type TG working mechanism for PTAm.

To assess the impact of Δ*S*_red_ on thermoelectric performance, the temperature dependence of the half-cell potential (*E*_half_) of water-soluble ox-PTAm was measured (Figure 1c) using the following equation derived from eq 1:

5 $$E_{\mathrm{half}}=T\frac{\Delta S_{\mathrm{red}}}{nF}-\frac{\Delta H_{\mathrm{red}}}{nF}$$

where Δ*H*_red_ represents the reduction enthalpy changes. The slope of the *E*_half_ versus *T* curve yields Δ*S*_red_ = 73.4 J K^–1^. From eq 3, the corresponding thermpower (α) is calculated to be −0.76 mV K^–1^, indicating the n-type thermoelectric behavior of PTAm. Figure 1c schematically illustrates the working principle of the PTAm-based n-type TG cell. Under a temperature gradient, the half-cell potential shift generates a voltage across the cell. According to Figure 1b, the potential increases with the temperature, indicating that the hot electrode works as the positive electrode (cathode, reduction) and the cold electrode as the negative electrode (anode, oxidation). During the operation, the oxoammonium cations are reduced at the hot side while TEMPO radicals are oxidized at the cold side, demonstrating bidirectional redox activity and establishing a thermoelectric voltage. This bidirectional redox activity generates the thermoelectric voltage, demonstrating that the coexistence of TEMPO and oxoammonium cation in the water-soluble ox-PTAm makes the polymer suitable for TG application. Moreover, the output performance of PTAm TG cell can be reinforced by fast reaction kinetics of TEMPO/oxoammonium cation redox couple, highlighting the potential of PTAm for low-grade heat harvesting applications.

The n-type TG behavior of water-soluble ox-PTAm can be rationalized by changes in the polymer end-to-end distance (*R*) during the redox reaction. In general, the *R* of a polymer chain is primarily influenced by intrachain interactions among its monomer units. In the case of ox-PTAm, the oxidized oxoammonium cation units introduce strong electrostatic repulsions between monomers, leading to an expansion of the polymer chain and an increase in the *R*. It is known to depend both on the ratio of nitroxide radical and oxoammonium cation and on the corporation ratio of TEMPO groups within the polymer chain.^37^ This change in chain conformation is associated with a decrease in conformational entropy (*S*_C_), which can be described by the following expression:

6 $$S_{C}\left(N,R\right)=-\frac{3}{2}k_{b}\frac{R^{2}}{Nb^{2}}+S_{C}\left(N,0\right)$$

where *N* is the degree of polymerization, *b* is the Kuhn length of polymer, and *k*_b_ is the Boltzmann’s constant. Notably, this equation indicates that a larger *R* corresponds to a lower *S*_C_. To validate the assumption of polymer conformational change upon redox reaction,^37–40^ the GFN force field simulation provides the precise insight into polymer conformation. Figure 2 shows the simulation results for 9-monomer PTAm and its oxoammonium-substituted counterpart, where the *R* values are 9.76 and 14.66 Å, respectively. It should be noted that the simulation was performed under isolated oligomer in vacuum and thus do not account for the ionic shielding effect from the counterion in aqueous electrolytes, which can potentially suppress the chain expansion after oxidation. Therefore, the calculation merely demonstrates the anticipated electrostatic chain expansion for the oxidized oligomer. The observed increase in *R* still qualitatively supports the anticipated chain expansion after oxidation. Moreover, the *R* of the ideal polymer chain can be estimated by *R* = *bN* ^1/2^, where the 9-monomer–oligomer PTAm shows a *R* of 8.22 Å. This suggests the feasibility of simulation results. Therefore, the oxidized state (oxoammonium state) represents a low entropy state while the reduced state (TEMPO state) corresponds to a high entropy state. This redox-coupled conformational change introduces an entropic contribution to the overall redox reaction, contributing to the observed n-type TG behavior of ox-PTAm, as shown in Figure 3a and Figure S6.

**Figure 2:**
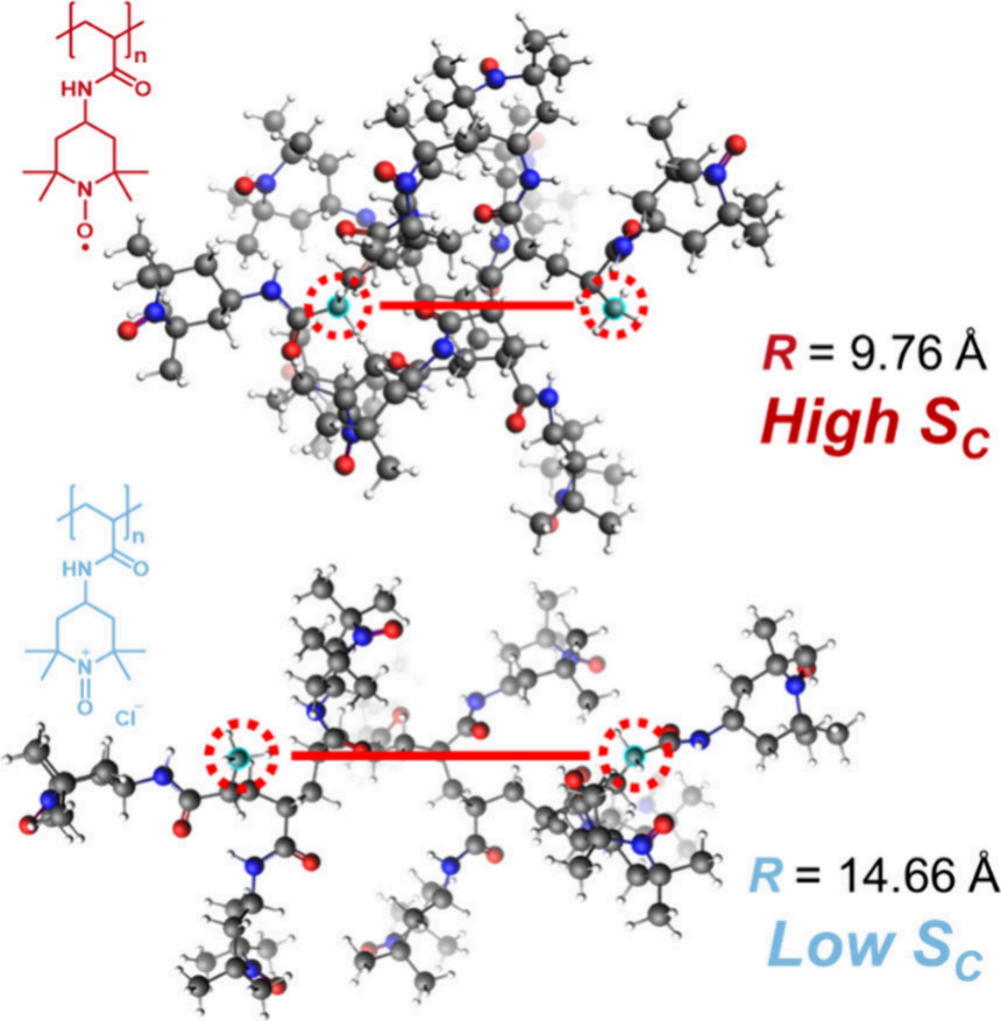
Simulated oligomers end-to-end distance (*R*) of PTAm (top) and oxoammonium-substituted polyacrylamide (bottom). Geometry optimizations are performed using the GFN force field.

**Figure 3:**
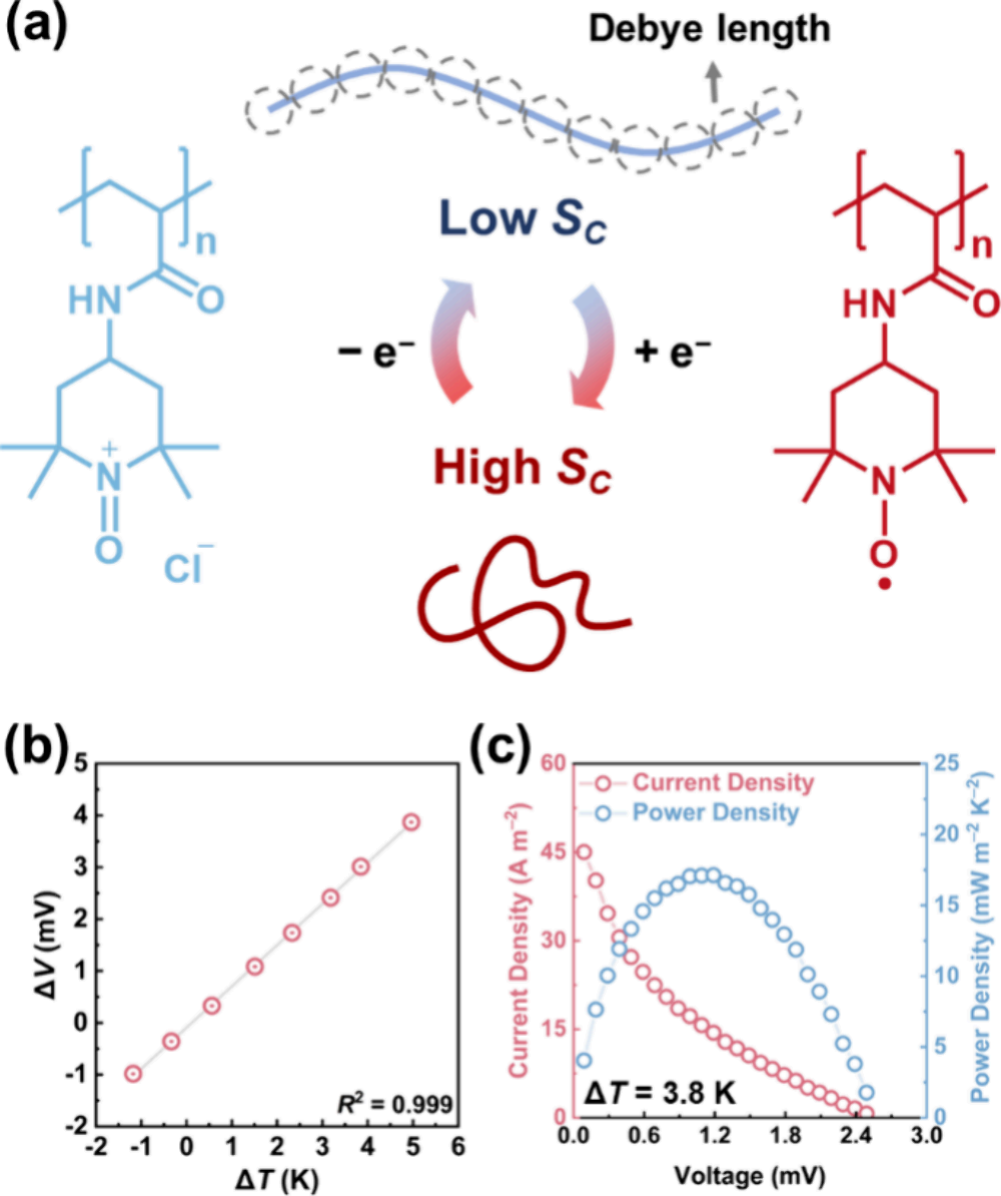
(a) Schematic illustration of the relationship between redox state and conformational entropy (*S*_C_) in water-soluble ox-PTAm. (b) Δ*V*–Δ*T* plot used to determine thermopower (α). (c) Power density measurement obtained by linear scan voltammetry (LSV) under an applied temperature gradient of 3.8 K.

To evaluate the thermoelectric performance of water-soluble ox-PTAm, a temperature gradient is applied to a thermocell, and the open-circuit voltage (Δ*E*) is recorded as a function of the temperature difference (Δ*T* = *T*_hot_ – *T*_cold_). As shown in Figure 3b, the measured thermopower (α) is – 0.76 ± 0.02 mV K^–1^, consistent with the value derived from electrochemical entropy measurements. This agreement further supports the interpretation that α can be manipulated via *S*_C_ associated with the redox process, confirming the n-type character of the water-soluble ox-PTAm. Although the measured α is slightly lower than that of conventional n-type redox couples such as Fe^2+/3+^, the exceptional reaction kinetics of the TEMPO/oxoammonium cation redox couple compensate by enabling higher output current densities. The maximum power output normalized by the square of the temperature gradient, was determined via linear sweep voltammetry (LSV) to be *P*_max_/Δ*T* ^2^ = 1.18 ± 0.01 mW m^–2^ K^–2^ at 3.8 K (Figure 3c). These results demonstrate that the PTAm-based system combines moderate thermopower with fast charge-transfer kinetics, offering compelling promise for thermogalvanic harvesting of low-grade heat.

In summary, we report the successful synthesis of low molecular weight TEMPO-substituted polyacrylamide (PTAm). Although the as-prepared PTAm is water-insoluble, electrochemical oxidation of the TEMPO moieties to oxoammonium cations transforms the neutral polymer into a water-soluble polyelectrolyte. The ox-PTAm retains excellent redox activity, as evidenced by CV. These fast reaction kinetics are advantageous for efficient charge transport in TG applications. Furthermore, we demonstrate that redox-induced conformational changes in PTAm result in *S*_C_, which enables n-type TG behavior. Thermoelectric characterization under an applied temperature gradient reveals a thermopower of −0.76 ± 0.02 mV K^–1^ and a power output of *P*_max_/Δ*T* ^2^ = 1.18 ± 0.01 mW m^–2^ K^–2^, in good agreement with electrochemically derived values. These findings highlight the potential of redox-active conformation-responsive polymers as a promising platform for next-generation TG materials designed for low-grade heat harvesting.

## Supplementary Material



## Notes

Experimental method, characterization of PTAm, EIS of ox-PTAm, and a schematic illustration of the ox-PTAm thermogalvanic device (PDF)
