# Supplementary material for: Designing n‑Type Thermogalvanic TEMPO-Substituted Polyacrylamide via Conformational Entropic Modulation
Source: ACS Macro Lett. 2026 Jan 22;15(2):282–7. doi: 10.1021/acsmacrolett.5c00762 (PMC12918710; doi:10.1021/acsmacrolett.5c00762)
Supplement: Supplementary file 1 [file mz5c00762_si_001.pdf]

Supporting Information

Designing n-Type Thermogalvanic TEMPO-Substituted Polyacrylamide via Conformational Entropic Modulation

Ching-Chieh Hsu,[a] *Kohei Ishigami,*[b] *Ryo Shirakawa,*[b] *Hiroyuki Nishide,*[a,c] *Kenichi Oyaizu,*[b,c*] *Cheng-Liang Liu*[a,d,e*]

[a] Department of Material Science and Engineering, National Taiwan University, Taipei 10617, Taiwan.

[b] Department of Applied Chemistry, and Research Institute for Science and Engineering, Waseda University, 3-4-1 Okubo, Shinjuku, Tokyo, 169-8555, Japan

[c] Research Institute for Science and Engineering, Waseda University, 3-4-1 Okubo, Shinjuku, Tokyo, 169-8555, Japan

[d] Institute of Polymer Science and Engineering, National Taiwan University, Taipei 10617, Taiwan.

[e] Advanced Research Center for Green Materials Science and Technology, National Taiwan University, Taipei 10617, Taiwan.

KEYWORDS: TEMPO, polyacrylamide, themogalvanic cell, electrochemistry, redox

Experiment method

Synthesis of *N*-(2,2,6,6-tetramethylpiperidin-4-yl) Acrylamide (TAm). 4-amino-2,2,6,6-tetramethylpiperidine (13 mL, 7.48 mmol) was firstly added into a round bottom flask, which contains 100 mL DCM and a stir bar, resulting a slightly yellow and clear solution. The solution was continuously stirred in an ice bath and followed by adding acryloyl chloride (2.4 mL, 296 mmol) dropwisely into the solution under nitrogen atmosphere. The reaction was held for 1 hour with continuously stirring. The solution was washed with saturated aqueous sodium carbonate, dried over anhydrous magnesium sulfate, and concentrated under reduced pressure to remove DCM. The residual solution was recrystallized in hexane/ethanol, resulting a white solid. The white solid was then dried in vacuum overnight. The product yield is around 79.4%. The ^{1}H NMR shows δ 6.28 (1H, d), δ 6.06 (1H, q), δ 5.63 (1H, dd), δ 5.29 (1H, s), δ 4.35 (1H, d), δ 1.94 (2H, dd), δ 1.27 (6H, s), δ 1.13 (6H, s), δ 0.94 (2H, t).

TAm Polymerization. TAm (1.05 g, 5 mmol) was added into 5 mL round bottom flask with a stir bar. 4 mL DMF was then added into the flask and heated in 65°C for 20 minutes with nitrogen bubbling to evacuate the oxygen in the solution. A solution of AIBN (16.5 mg, 0.1 mmole) in 1 mL DMF was directly added into the flask to initiate the polymerization. After 20 hours, the viscos mixture was diluted by methanol and added dropwisely into conical flask contained 200 mL of 2:1 hexane/EtOAc solution with vigorous stirring. After removing the solvent, the white solid was dried in vacuum oven with 80°C overnight. The yield is around 80%. The ^{1}H NMR shows 6.42 (1H, br s), δ 5.29 (1H, br s), δ 4.18 (1H, br s), δ 2.80-1.55 (5H, br m), δ 1.25 (6H, br s), δ 1.12 (6H, br s), δ 1.00 (2H, br s).

Oxidation of Piperidine-substituted Polymer. 200 mg of the piperidine-substituted polymer was dissolved in 1 mL DMF with ice water bath. Another solution of 4.26 g mCPBA in 4 mL DMF was slowly added into the polymer solution. After 1 hour, the red-orange mixture was added dropwisely into the conical flask contained 200 mL of 2:1 hexane/EtOAc solution with vigorous stirring. The red precipitates were further washed with 2:1 hexane/EtOAc solution to remove more DMF. The precipitate was then dried in 80°C vacuum oven overnight. the yield of PTAm is around 95%.

Water-soluble Oxidized PTAm (ox-PTAm). A carbon cloth was cleaned by sequential ultrasonication in deionized water, acetone, and ethanol, each for 15 minutes in a water-bath sonicator. 100 µL of 200 mg mL^{-1} PTAm solution in THF was directly drop onto the cleaned and dried carbon cloth with surface area of 1 cm×cm. The PTAm-coated electrode was then dipped into a U-type cell, with the alignment of PTAm | 3 M $KCl_{(aq)}$ ‖ 3 M $KCl_{(aq)}$ | Pt. The volume of anolyte and catholyte were both 8 mL. To further increase the PTAm concentration in water, 4 PTAm-coated electrodes in total were used in the cycling and each electrode was cycled 50 times with scan rate of 100 mV s^{-1}. After cycling of each electrode, the solution starts to turn yellower. The water-soluble PTAm was the isolated by dialysis and lyophilization. The concentration of the water-soluble PTAm solution was estimated around 2 mg mL^{-1}.

Polymer characterization. 1H-NMR (500 MHz) spectra were recorded using JEOL-ECX500. A Quantum Design MPMS SQUID-VSM magnetometer was used for magnetization measurements. Size exclusion chromatography (SEC) was performed by SHIMADZU LC-20AD/CBM-20 A with the attached TOSOH TSKgel SuperAW5000 column and SHIMADZU SPD-M20 A UV detector (dimethylformamide (DMF) with 10 mM LiCl, flow rate: 0.3 mL min^{-1}, UV detection wavelength: 275 nm (DMF), calibration reference: polystyrene standards).

Electrochemistry. All electrochemical measurements were carried out using a BioLogic SP-150e potentiostat. Platinum (Pt) electrode served as the working and counter electrode, with dimensions of 2 mm ×5 mm and 15 mm × 10 mm respectively, while an Ag/AgCl (3 M KCl) electrode was used as the reference. For CV of water-soluble PTAm, the potential was swept from – 0.3 V to 0.9 V vs. Ag/AgCl (3 M KCl) across all samples at scan rates ranging from 200 mV s^{-1} to 400 mV s^{-1}. For EIS, the data were recorded over a frequency range of 1 MHz to 50 mHz using an AC perturbation of 10 mV without overpotential, and fitted to a Randles circuit model using ZFit software.

Thermoelectric Measurement. The thermoelectric performance of the water-soluble ox-PTAm was characterized using a custom-designed measurement setup. A PDMS holder, with dimensions of 20 mm × 20 mm × 3 mm, was sandwiched between two Pt electrodes, The center of PDMS have a hole with diameter of 4 mm and the water-soluble PTAm solution was filled into the cavity. The electrodes were affixed directly to the heat sinks, with appropriate insulation applied to ensure reliable and accurate electrical contact. The distance between the two heat sinks was consistently maintained at 3 mm for all tests. The cold-side heat sink maintained at 20 °C via a water-cooling system and the hot side heated by a Joule heating unit. A Keithley 2400 source meter was employed to measure the open-circuit voltage (V_{OC}) generated across the hydrogel under various temperature gradients. All measurements were conducted in an environmental chamber controlled at 25 °C and 30% relative humidity (RH). The thermoelectric power measurement was done by linearly sweeping voltammetry (LSV) method using BioLogic SP-150e potentiostat. To determine the thermoelectric power, A temperature gradient was applied to the cell and the V_{OC} was determined. The LSV scan window ranged from V_{OC} to 0 mV, with scan rate of 0.2 mV s^{-1}

Computational Methodology. The 9-monomer oligomer was modeled using the AMS Polymer Builder. The simulation workflow involved a dual-stage optimization strategy: first, a coarse-grained refinement was executed via the Universal Force Field (UFF) to eliminate steric clashes and establish a stable backbone orientation. Subsequently, the structure was fully relaxed using the GFN-FF method, which offers a more sophisticated treatment of the electronic environment and non-covalent effects than traditional force fields. This two-step approach ensures that the resulting conformation represents a refined energy minimum suitable for calculating polymer conformation.

Supplementary Note S1. PTAm Characterization. To confirm the successful formation of the open-shell radical structure, electron spin resonance (ESR) spectroscopy and superconducting quantum interference device (SQUID) magnetometry were employed. As shown in Figure S1a, the ESR spectrum of PTAm exhibits a signal with a g-factor of 2.0065, assigned to the TEMPO radical. The broadening of the ESR signal is attributed to intrachain dipole–dipole interactions between closely spaced radical sites along the polymer backbone. Figure S1b presents the reciprocal magnetic susceptibility ($1/\chi$) as a function of temperature, measured by SQUID. The linearity of the $1/\chi$ versus T plot follows the Curie-Weiss law: $1/\chi_{\mathrm{paramagnetic}} = T/C - \theta/T$, where $C = Ng^2u_B{}^2S(S+1)/3k_\mathrm{b}$, N stands for the total number of unpaired electrons, the radical concentration (C) can be estimate by the slope of $1/\chi$ versus T. From the slope of the curve, the radical concentration is estimated to be approximately 83% relative to the total number of monomer units.

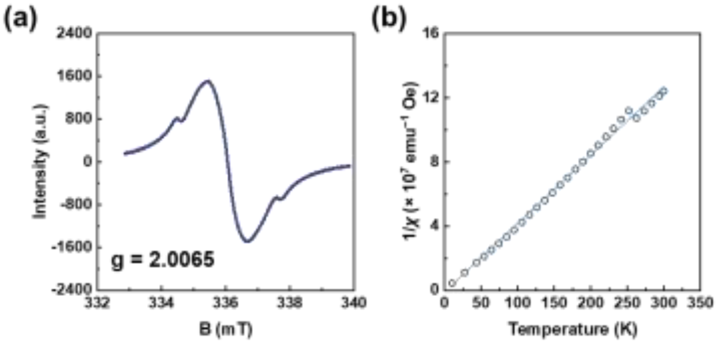

Figure S1. (a) Electron spin resonance (ESR) signal of PTAm polymer (monomer concentration of 1 μmol L^{-1} in THF) in DCM at room temperature. (b) Reciprocal of magnetic susceptibility ($1/\chi$) as a function of temperature, measured by superconducting quantum interference device (SQUID).

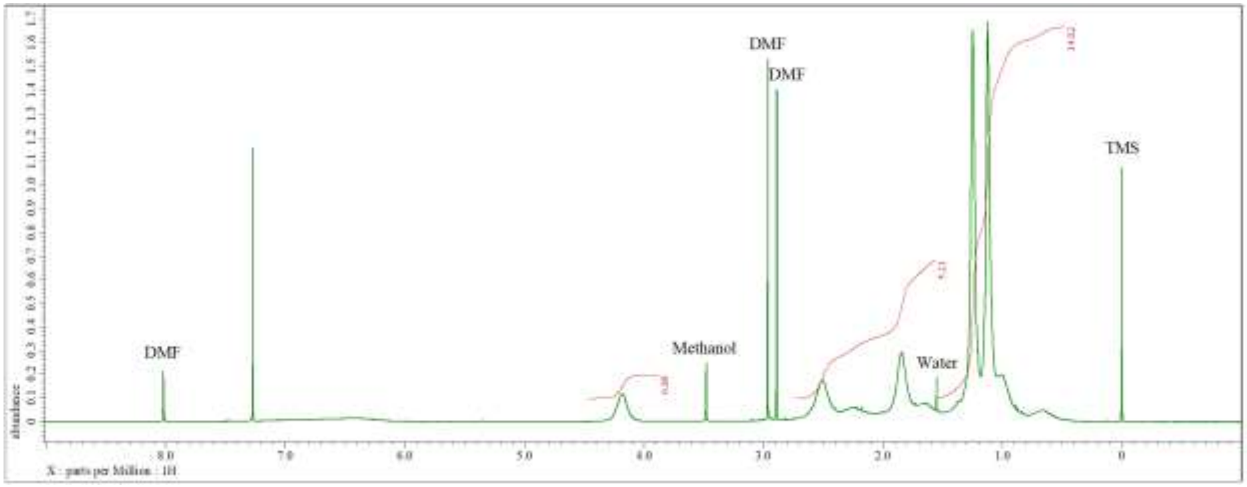

Figure S2. ^{1}H-NMR spectrum of PTAm precursor in $CDCl_3$.

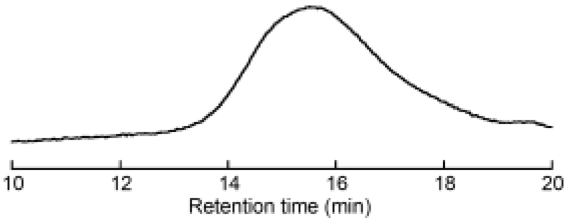

Figure S3. SEC chromatogram of PTAm in DMF containing 10 mM LiCl.

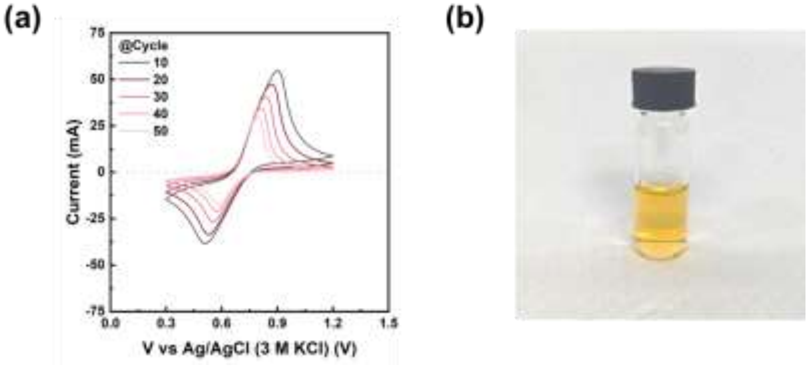

Figure S4. (a) Cyclic voltammetry (CV) profile of PTAm-coated carbon cloth in a U-type cell (PTAm | 3 M $KCl_{(aq)}$ ‖ 3 M $KCl_{(aq)}$ | Pt). (b) The picture of anolyte, the yellow color is attributed to the water-soluble ox-PTAm.

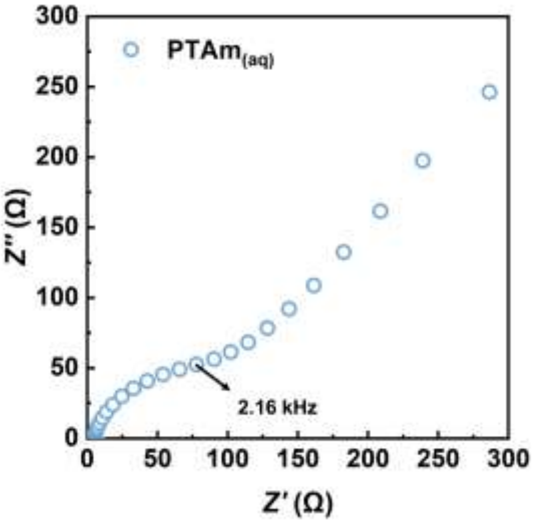

Figure S5. Electrochemical impedance spectroscopy (EIS) of water-soluble ox-PTAm, with AC frequency ranging from 215 kHz to 147 mHz.

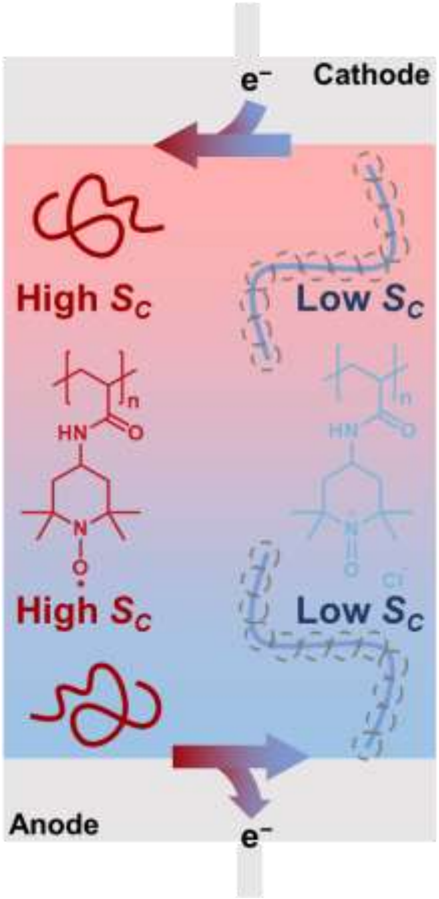

Figure S6. Schematic illustration of an ox-PTAm thermogalvanic device.
